# Adaptor Template Oligo-Mediated Sequencing (ATOM-Seq) is a new ultra-sensitive UMI-based NGS library preparation technology for use with cfDNA and cfRNA

**DOI:** 10.1038/s41598-021-82737-9

**Published:** 2021-02-04

**Authors:** Thomas L. Dunwell, Simon C. Dailey, Anine L. Ottestad, Jihang Yu, Philipp W. Becker, Sarah Scaife, Susan D. Richman, Henry M. Wood, Hayley Slaney, Daniel Bottomley, Xiangsheng Yang, Hui Xiao, Sissel G. F. Wahl, Bjørn H. Grønberg, Hongyan Dai, Guoliang Fu

**Affiliations:** 1 GeneFirst Ltd, Building E5, Culham Science Centre, Abingdon, OX14 3DB UK; 2 Pathology and Data Analytics, Leeds Institute of Medical Research, University of Leeds, St James University Hospital, Leeds, LS9 7TF UK; 3 Guangzhou Biotron Technology Co., Ltd, Room 204, Zone C, Science and Technology Innovation Base, No. 80, Lanyue Road, Science City, Guangzhou, China; 4 Department of Oncology, St. Olav’s Hospital, Trondheim University Hospital, Trondheim, Norway; 5 Department of Clinical and Molecular Medicine, Faculty of Medicine and Health Sciences, Norwegian University of Science and Technology (NTNU), Trondheim, Norway; 6 Department of Pathology, St. Olav’s Hospital, Trondheim University Hospital, Trondheim, Norway

**Keywords:** Biological techniques, Cancer, Genetics

## Abstract

Liquid biopsy testing utilising Next Generation Sequencing (NGS) is rapidly moving towards clinical adoption for personalised oncology. However, before NGS can fulfil its potential any novel testing approach must identify ways of reducing errors, allowing separation of true low-frequency mutations from procedural artefacts, and be designed to improve upon current technologies. Popular NGS technologies typically utilise two DNA capture approaches; PCR and ligation, which have known limitations and seem to have reached a development plateau with only small, stepwise improvements being made. To maximise the ultimate utility of liquid biopsy testing we have developed a highly versatile approach to NGS: Adaptor Template Oligo Mediated Sequencing (ATOM-Seq). ATOM-Seq's strengths and versatility avoid the major limitations of both PCR- and ligation-based approaches. This technology is ligation free, simple, efficient, flexible, and streamlined, and it offers novel advantages that make it perfectly suited for use on highly challenging clinical material. Using reference and clinical materials, we demonstrate detection of known SNVs down to allele frequencies of 0.1% using as little as 20–25 ng of cfDNA, as well as the ability to detect fusions from RNA. We illustrate ATOM-Seq’s suitability for clinical testing by showing high concordance rates between paired cfDNA and FFPE clinical samples.

## Introduction

The ever-expanding use of next generation sequencing (NGS) has been instrumental in exploring and understanding the changes which occur during cancer development. This has revealed expansive patterns of single-nucleotide variants (SNVs), insertions, deletions, copy number variations (CNVs) and gene fusions as frequently occurring events across one or more cancer types, an approach epitomised by studies such as The Cancer Genome Atlas^1^. The understanding this has given about cancer development has driven the development of both PCR- and NGS-based technologies for use in the detection of these cancer-associated changes. Historically these technologies and associated tests have focused on identifying mutations through testing of tumour biopsies, and with these materials previous technologies have performed well. Testing of primary tumours is relatively simple, but obtaining this material can be extremely invasive. In an attempt to improve patient outcomes, attention has therefore turned towards using minimally invasive liquid biopsies. Testing this material is a highly challenging approach which is motivated by the fact that cancer-related biomarkers can be detected in these easily obtained blood samples without any prior knowledge of the location of a tumour and potentially at a far earlier stage of disease progression.

There are wide ranging potential benefits to liquid biopsy testing, as these samples can contain a combination of cell-free DNA and (to a lesser extent) cell-free RNA (cfDNA/cfRNA) derived from primary tumours; clinical testing of these molecules allows for the indirect detection and genotyping of tumours (including SNV/CNV/fusions). The ability to interrogate these samples holds great promise for early diagnosis and for disease monitoring in relation to determining the success of targeted therapies and detecting the emergence of drug resistance clones, as well as in assessing disease recurrence and minimal residual disease monitoring. Unfortunately, the majority of the material in a liquid biopsy is derived from non-cancerous cells. As a result, DNA/RNA from a tumour may only be present at a very low proportion (0.01%) of the total quantity of nucleic acids in a sample. This poses a significant challenge as the apparent allele frequency of the mutation may fall below the background error rate for a given technology. Successfully demonstrating clinical validity and clinical utility of testing liquid biopsy-derived material using NGS will therefore depend on the availability of technologies that are able to detect cancer-associated changes with very high sensitivity and specificity^2^.

When considering the NGS technologies currently available, these can be broadly categorised as ‘whole genome’ or ‘targeted’ approaches. The depth necessary to identify rare and ultra-rare variants (< 0.5–1%) makes whole genome sequencing costly and impractical, and as such the majority of commercially adopted approaches have focused on targeted enrichment technologies for cancer-associated mutation testing. The detection of low frequency mutations by these target enrichment processes are hampered by a low level of background errors, which are present in all workflows^3–7^. To combat this, technologies have been adapted to incorporate unique molecular identifiers (UMI). These are short regions of nucleic acids that are added to original DNA fragments, or copies thereof, in the first step(s) of an enrichment protocol. All subsequent PCR copies will share the same unique UMI, meaning these sequences can be used to group and de-multiplex all sequencing reads deriving from a single original molecule. As a result, many artefactual amplification errors (such as mis-incorporation of nucleotides by polymerases) can be corrected generating consensus reads accurately representing the original starting material.

These UMI-containing enrichment technologies can broadly be separated between (1) those approaches which use PCR to directly amplify the original DNA^8^, and (2) those based on ligation of adaptors onto the ends of DNA. The latter approach can be further subdivided into those which either undergo subsequent probe based capture^9,10^ or PCR-based enrichment using ligation products as a template^11–13^. Both methods have been utilised in the development of products targeted towards testing clinical samples for ultra-rare variants, especially with liquid biopsy samples. Cell-free DNA extracted from liquid biopsy samples is enriched in short (~ 171 bp), single-strand, and damaged molecules^14–16^ and this results in issues for the aforementioned technologies. The reliance of purely PCR-based approaches on a predetermined pair of opposing primers means they are ill-suited to identifying large indels and translocations or to heavily fragmented input material. These approaches also cannot detect unknown gene fusions. Ligation-based approaches used in targeted enrichment protocols generally focus on the ligation of a double-strand adaptor onto end-repaired dsDNA, as such these approaches are not optimised to work with single-strand DNA and may also struggle with damaged DNA. Approaches have been developed with an eye towards improving efficiency with ssDNA^17–19^, however there are not yet any commercially available products targeting low frequency mutation detection based on these approaches. In addition, ligation-hybrid capture approaches struggle with capturing short DNA fragments and may also have very long and complex protocols. The inherent limitations of the aforementioned technologies in processing liquid biopsy samples means they cannot maximise their potential sensitivity in detecting cancer-associated changes obtained from such clinical samples. As such there is a need for the development of new approaches which are specifically designed for liquid biopsy testing and are focused on overcoming these limitations.

With both the limitations of current technologies and biological context of cell-free nucleic acids in mind, we re-imagined the process of library preparation and developed a novel technology designed specifically for use with challenging clinical material such as cfDNA/cfRNA obtained from liquid biopsies: **A**daptor **T**emplate **O**ligo-**M**ediated **Seq**uencing—ATOM-Seq.

In this paper we describe ATOM-Seq’s technological approach for NGS library preparation and detail its highly efficient, robust and rapid protocols, showing its suitability for targeted library preparation methods using RNA or DNA as starting material. We demonstrate the technology’s sensitivity with reference material of up to 100% for detecting very low frequency variants (~ 1%) with 5 ng of fragmented DNA. We demonstrate sensitivities of 94% for rare variants down to 0.1% allele frequency using as little as 20 ng of fragmented DNA, and also demonstrate identification of mutations down to 0.1% allele frequency using 20–25 ng of cfDNA. We also demonstrate specificities, by using an inferred ground truth, for 1% variants of 97.3%. With clinical samples we report excellent performance on colorectal cancer formalin-fixed paraffin-embedded (FFPE) samples and on lung cancer liquid biopsy samples and FFPE samples, when compared with previous technologies. With paired lung cancer FFPE and liquid biopsy samples we demonstrate concordance rates > 83% for de novo mutation detection. With RNA we demonstrate ATOM-Seq’s suitability for detecting gene fusions, and with total nucleic acid samples (combined cfRNA and cfDNA) we demonstrate an ability to capture and enrich for both cfRNA and cfDNA molecules in a single reaction.

## Results

### Adaptor Template Oligo Mediated Sequencing: ATO reaction

The main goal of this project was to design a new approach for NGS-based liquid biopsy material testing. To achieve this, we developed a new NGS library preparation technology named **A**daptor **T**emplate **O**ligo **M**ediated **Seq**uencing—ATOM-Seq. This technology was designed to be compatible with any suitable starting material including single- and double-strand DNA, cfDNA, enzymatically fragmented or sonicated gDNA or FFPE DNA, and single- and double-strand cDNA produced from FFPE- or cell-free-derived RNA.

**Figure 1:**
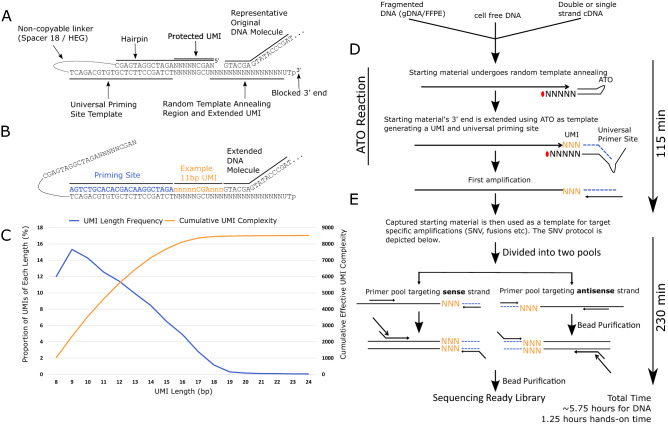
Overview of ATOM-Seq approach and targeted enrichment protocol. ATOM-Seq based protocols can take any suitable starting material including cell-free DNA, fragmented FFPE DNA, or cDNA. (**A**) A detailed overview of an ATO and its annealing to a representative DNA molecule. ATOs consist of a blocked 3′ end preceded by a U nucleotide, a 15 bp string of Ns to act as the random annealing site and as part of the UMI, a double strand stem region containing a protected 8 bp UMI and a universal priming site. The two strands of the stem are linked by a non-DNA spacer. (**B**) A representative product of an ATO Reaction where a DNA molecule was extended on an ATO template, generating an 11 bp UMI. (**C**) In blue, a frequency distribution analysis of UMI length, starting from the minimum 8 bp protected UMI. In orange, the cumulative complexity of the random region generated during the 3′ extension reaction, taking into consideration the increasing potential complexity but declining frequency of longer UMIs. (**D**) Protocol overview. The first step is an “ATO Reaction” which captures the starting material by extending their 3′ ends using an ATO as a template, generating a Unique Molecular Identifier and a universal primer site. (**E**) Target enrichment is accomplished by dividing the first amplification into two separate pools for two rounds of nested PCR. Each pool targets either the sense or antisense DNA strands allowing both strands to be independently enriched.

The underpinning conceptual idea of ATOM-Seq was to use the original sample DNA as a primer and extend its free 3′ end, thereby allowing ‘capturing’ of every molecule in the starting material. This was accomplished by designing an approach where the starting material is combined with a synthetic oligo, termed an Adaptor Template Oligo (ATO; Fig. 1A). The 3′ ends of the starting material anneal to a single-strand random sequence contained on the 3′ end of the ATO. Through the addition of a suitable polymerase the starting material then functions as a primer and is extended using the ATO as a template (Fig. 1B). This 3′ extension reaction copies the DNA sequence from the ATO onto the 3′ end of the starting material, ‘capturing’ the DNA or cDNA ends. During this reaction two separate functional components are created on each 3′ end. The first is a universal primer site which is used during downstream amplification reactions. The second is a random sequence which functions as a true Unique Molecular Identifier (UMI), allowing for bioinformatic error correction after sequencing. The UMI is separated into two regions: a protected UMI, which is contained within the double strand stem region of the oligo, and the extended UMI, whose length is dependent on the random annealing position of the original DNA. The incorporation of these elements onto the original molecule—a process which we have termed ‘ATO Reactions’—produces molecules which are conceptually similar to those generated by traditional UMI-based ligation approaches such as anchored multiplex amplification^11^ and single primer extension^12^.

To investigate the complexity of the UMIs generated during an ATO Reaction, a pool of single strand DNA oligos were used as starting material with a whole sample protocol. Due to the random nature of the UMIs this approach is necessary, as a well-defined, fixed series of sequences must be used in order to precisely separate input sequence and UMI sequence. This demonstrated that UMI lengths were up to 19 bp, with the most frequent length being 9 bp (Fig. 1C; Supplementary Table 1). As the frequency of UMIs of each length drops as the UMI length grows beyond 9 bp, only a small fraction of the total theoretical complexity of the random region is utilised. The effective complexity was determined by first identifying the UMI length whose complexity would saturate first, this was the shortest 8 bp UMI which has a maximum complexity of 4^5^ or 1024 variations. The frequency of all UMI lengths was calculated relative to this 1024. As an example, ~ 15% of UMIs were 9 bp relative to ~ 12% which were 8 bp so there were 25% more, for a total of 1300 9 bp UMIs for every 1024 8 bp UMI. Summing across all the UMI lengths gives a total effective complexity of 8,500 different UMIs for every unique 3′ DNA end (Fig. 1C). This indicates that UMIs will be in great excess relative to randomly fragmented DNA molecules in any ATO Reaction.

### Targeted enrichment of captured DNA

To follow on from the capture of starting material by an ATO Reaction, we developed a target enrichment protocol that is designed to maximise both sensitivity and specificity. The captured material undergoes an initial linear amplification, which uses a universal primer complementary to the universal priming site at the 3′ of each starting DNA molecule (Fig. 1D). Repeated rounds of this linear amplification create multiple copies of each original starting molecule. After this, separate workflows were designed depending on the purpose of the library preparation. The first workflow is for targeted enrichment of frequently mutated regions from DNA (Fig. 1E); the second is for known and unknown fusion detection from RNA input (Supplementary Fig. 1), the third is whole sample (a.k.a. whole genome) library preparation (Supplementary Fig. 2).

For targeted PCR enrichment-based detection of mutations, the first linear amplification product is split into two halves, each acting as a template for separate pools of target-specific primers. This design allows for two primer pools to independently amplify either the sense or antisense DNA strands of the original starting material, in combination with a universal primer, while eliminating the risk of opposing primers interfering with one another. Strand-specific targeting is possible as a target-specific primer can only exponentially amplify a DNA molecule if it forms a correctly orientated pair with the universal primer. Following this first round of PCR is a second round of nested PCR enrichment, which also incorporates dual sample indices (Fig. 1E).

The variation of this protocol for detecting the presence of known and unknown fusions from an RNA sample is very similar to the above, comprising of two rounds of target-specific enrichment using nested primers. In this instance however, cDNA is generated prior to the ATO Reaction (Supplementary Fig. 1A,B), and the first amplification product is not split between pools of target-specific primers (Supplementary Fig. 1C). The whole sample version of this protocol uses the first amplification product as input for a second ATO Reaction (Supplementary Fig. 2B), which incorporates a second universal priming site at the opposite end of the captured molecule, allowing global PCR amplification of captured molecules (Supplementary Fig. 2C).

### Performance of SNV detection protocol with mutation reference standards

**Figure 2:**
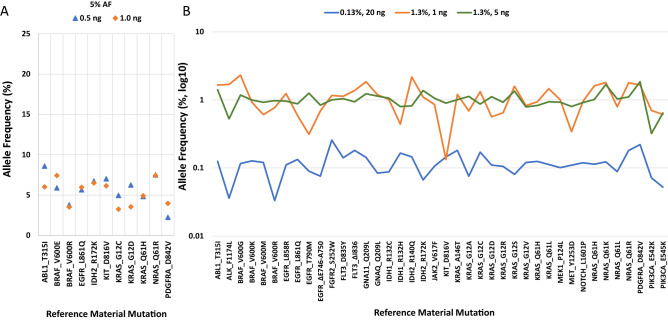
Technical validation of ATOM-Seq technology using reference standards. The plots show the average AF detected for each mutation in the reference material across 3 biological replicates for each of the starting DNA quantities for (**A**) **5%** AF with 0.5 ng and 1 ng, for which the variance of the average allele frequency was 3.5 and 2.17 respectively, and (**B**) **1.3%** AF with 1 ng and 5 ng with a variance of the average allele frequencies of 0.27 and 0.075 respectively, and **0.13%** AF with 20 ng with a variance of the average allele frequencies of 0.002. The reference material contains variants with allele frequencies > 5% or > 1.3%, which were detected but not shown.

In order to thoroughly evaluate the performance of the ATOM-Seq technology, a series of commercially available reference standards were used in combination with a large panel of approximately 1150 target enrichment primers designed to amplify approximately 575 regions across 100 genes frequently mutated in cancer with a total callable region of 59 kb. In order to assess sensitivity, standards with different expected allele frequencies (AF) and input quantities were used. A 5% AF standard was used with both 0.5 ng and 1.0 ng of material, a 1.3% AF standard was used with both 1.0 ng and 5.0 ng, and a 0.13% AF standard was used with 20 ng. An overview of the sequencing data and sensitivity is shown in Fig. 2 and Table 1. Detailed sequencing information of the biological replicates is in Supplementary Tables 2–5.

**Table 1:** Sequencing quality and sensitivity of ATOM-Seq SNV protocol on reference samples.

	AF:	5.0%		1.3%				0.13%
	Input (ng):	0.5	1.0	1.0		5.0		20.0
Average sequencing depth per primer	Sense DNA	395	807	870		2193		9361
	Antisense DNA	426	973	1147		2448		9759
	Combined	820	1780	2017		4640		19,119
Average primer uniformity	Sense DNA	95.7%	96.1%	96.4%		96.8%		96.5%
	Antisense DNA	94.0%	94.3%	94.7%		94.3%		95.1%
	Combined	94.9%	95.2%	95.6%		95.5%		95.8%
Average primer on-target rate	Sense DNA	84.5%	85.6%	84.4%		83.9%		84.4%
	Antisense DNA	90.8%	90.6%	91.1%		90.5%		89.6%
	Combined	87.7%	88.1%	87.7%		87.2%		87.0%
Sensitivity of reference mutations detected across all 3 replicates		100%	100%	90.2%		100%		94.0%

Genome mapping rates were > 99% for all samples.

With both 0.5 ng and 1.0 ng of reference material DNA containing 5% AF mutations, a total of 100% of expected variants were detected in all three replicates. When using 1 ng of DNA with 1.3% AF, on average 90% of expected mutations were in each of the three replicates and every expected mutation was found in at least one replicate. With 5 ng of DNA at 1.3% AF, 100% of the expected variants were detected in all 3 replicates. To test the absolute sensitivity of ATOM-Seq, the AF was reduced further to 0.13% and the input DNA quantity was increased to only 20 ng, proportionally 2.5 × fewer copies of mutated material than used for 5 ng at 1.3% AF. In this instance, on average 94% of mutations were found in each of the three replicates and again all of mutations were identified in at least one replicate (Fig. 2).

The sensitivity and specificity of the ATOM-Seq protocol was examined with a semi-blind approach by first generating an ‘inferred ground truth’. This was accomplished by first combining the lists of variants found in each of the three reference material replicates where 5 ng of starting material was used. These combined samples were filtered to contain only those variants which fell within our target regions and had a variant count ≥ 3. This generated a list of 117 variants for replicate 1 and 118 variants for replicates 2 and 3. Of these, a total of 117 were identified as ‘inferred true positives’, defined as those variants observed in either 2 or 3 of the 3 samples (Supplementary Fig. 3A). As summarised in Table 2, for all variants, we found that average inferred true positive specificity was 98.3% and average sensitivity was 99.2%. These variants were then filtered further to contain those with low allele frequencies (≤ 2% AF). This reduced the list of variants to 49 inferred true positives (Supplementary Fig. 3B). With these very low frequency mutations, average specificity dropped slightly to 97.3% and sensitivity to 97.9%. With this data we were able to identify ‘inferred false positives’, with between 0 and 3 per sample for averages of 1.7 and 1.3 (Supplementary Fig. 3A,B, respectively). With a target region of approximately 59 kb this allows us to calculate an average of ~ 23–30 inferred false positives per megabase when using an off-the-shelf variant caller. To determine whether any apparent inferred true or false positives may in fact be systemic errors within the protocol, these variants were compared against the variants identified in clinical FFPE and cfDNA samples previously tested. Except for expected known mutations (such as deletions in EGFR) and SNPs, there was no evidence of variants common to all datasets that would be indicative of protocol or procedural artefacts.

**Table 2:** Sensitivity and specificity of ATOM-Seq SNV protocol on reference samples using 5 ng of starting material.

Replicate	Variant depth	All variants				Very low frequency variants			
		Total variants	Inferred true positives (n = 117)	Inferred true positive specificity	Inferred true positive sensitivity	Total variants	Inferred true positives (n = 49)	Inferred true positive specificity	Inferred true positive sensitivity
1	≥ 3	118	117	99.2%	100.0%	50	49	98%	100.0%
2	≥ 3	118	115	97.5%	98.3%	50	47	94%	95.9%
3	≥ 3	117	116	99.2%	99.2%	48	48	100.0%	98.0%
Average		118	116	98.3%	99.2%	49	48	97.3%	97.9%

### Performance of ATOM-Seq with FFPE and liquid biopsy DNA samples

In order to clinically validate the ATOM-Seq technology, three small pilot studies were completed. These studies used cancer samples which had previously been assessed for mutations by one or more alternative approaches. Studies were performed blind with the expected results only shared upon completion of the sequencing analysis. An overview of all sequencing and identified variants is available in Supplementary Tables 6–9.

**Figure 3:**
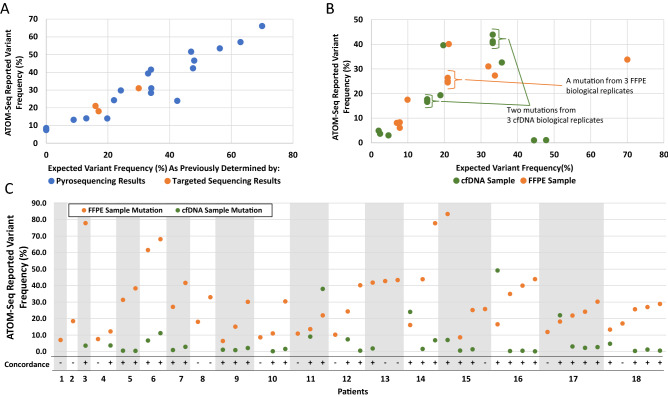
Technical validation of ATOM-Seq on clinical samples. (**A**) Colon cancer samples. Correlation of the allele frequencies reported by ATOM-Seq relative to those determined either by pyrosequencing (blue spots) or targeted sequencing (orange spots). ATOM-Seq showed superior sensitivity by detecting variants in two samples which were not found when analysed by pyrosequencing as these were below the technology's minimum sensitivity. (**B**) Lung cancer samples. Correlation of the allele frequencies reported by ATOM-Seq relative to those determined by an alternative NGS approach. (**C**) Paired lung cancer samples. Details of the allele frequencies for mutations found in FFPE samples and the concordant mutations from a matched cfDNA sample. A total of 84% (15/18) of patients had concordant mutations detected.

The first of these pilot studies used colon cancer samples and a panel of primers targeting mutations common in this cancer. A total of 14 of 20 FFPE samples were successfully processed and sequenced. These samples had been previously assessed for the presence of *KRAS*, *BRAF*, *NRAS* or *PIKC3A* mutations using pyrosequencing, or for selected *TP53* mutations with targeted PCR and sequencing. Upon comparison of the data generated by ATOM-Seq to the expected data, all the previously expected mutations were identified. Further, due to the higher sensitivity of ATOM-Seq, two additional *PIK3CA* mutations were identified which were below the sensitivity threshold of pyrosequencing (Fig. 3A), we also identified an *NRAS* variant which had been called as two separate variants by pyrosequencing was in fact a complex CC- > AG variant.

The second pilot study used a different panel of primers to identify mutations in unpaired lung cancer FFPE and cfDNA patient DNA samples. All samples were successfully processed, 7 cfDNA samples and 6 FFPE samples, all of which had previously been assessed for mutations by a third party with an in-house developed hybrid capture approach. From these, one sample of each material type was also processed in triplicate, to assess reproducibility. All of the previously identified mutations were identified by ATOM-Seq. In the FFPE samples a total of 9 expected mutations were identified (AF 6.1–40.1%) and in the cfDNA samples 10 expected mutations were identified (AF 1.0–41.2%). For those samples processed in triplicate, all expected mutations were observed at consistent allele frequencies (Fig. 3B).

The third pilot study used a larger 100-gene pan cancer panel for identifying mutations from a series of 20 paired lung cancer FFPE and cfDNA samples. All samples were successfully processed and sequenced, no mutations were identified in two FFPE samples. For the remaining 18 paired samples we assessed the concordance between the FFPE and cfDNA samples. A total of 49 mutations were identified in the FFPE samples and of these 37 were identified in a paired cfDNA sample (Fig. 3C). The allele frequency of the concordant mutations ranged between 0.13–49.1% for cfDNA and 6.38–83.3% for FFPE samples (Fig. 3C). All 20 of the original FFPE samples had previously been sequenced using either an Illumina trusight panel or QIAseq Comprehensive Cancer Panel (Qiagen). Where the ATOM-Seq panel overlapped the same target genomic regions, it detected all but 2 of the previously identified FFPE mutations. In three instances the QiaSeq panel had also been used on the 3 cfDNA samples, identifying two concordant mutations, in comparison ATOM-Seq was able to identity eight concordant mutations (in samples 4, 9, and 18).

### Performance of ATOM-Seq with FFPE RNA and liquid biopsy total nucleic acid samples

**Figure 4:**
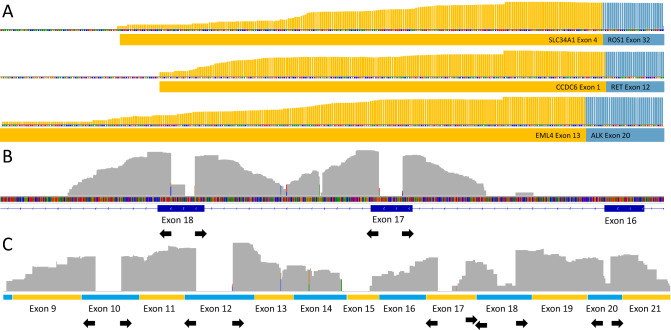
Technical validation of ATOM-Seq RNA based fusion protocol. (**A**) An FFPE fusion reference material containing 3 certified fusions was used to demonstrate fusion detection. Reads were mapped to contigs representative of the fusions, demonstrating reads spanning the EML4-ALK, CCDC6-RET, and SLC34A2-ROS1 fusions. (**B**,**C**) A total nucleic acid sample was used as starting material to the fusion protocol. (**B**) Shows DNA derived reads mapped to hg38 spanning exon–intron boundaries. (**C**) Shows reads which map to JAK1 (NM_001320923.1). The exons and orientation of the primers are shown, this demonstrates successful capture of cell-free RNA molecules as shown by the exon junction spanning reads. All read depths are shown in a log10 scale.

Next, we evaluated the performance of an ATOM-Seq based protocol designed for capturing RNA from disparate sources. To assess this, we used a commercial reference standard containing three validated fusions which we tested using a custom 170 primer panel with primers located in conserved fusion partners. This was designed to enrich targets known to frequently undergo fusion events in cancer and would be suitable for detection of both their known and unknown fusion partners. A total of 100 ng of FFPE RNA was used as starting material and the resultant library was sequenced to a depth of 0.8 million 75 bp PE reads, sequencing information in available in Supplementary Table 10. Upon analysis of the sequencing data the three expected fusion events were clearly identified (Fig. 4A).

To expand further we tested this protocol with a liquid biopsy total nucleic acid (TNA) extraction to assess the ability of the protocol to capture cell-free RNA along with the already validated cfDNA. A total of 13.65 ng of cell-free TNA were successfully processed and sequenced to a depth of 0.9 million 75 bp PE reads. Upon analysing the sequencing we were able to identify successful capture of both DNA and RNA molecules from within a single cell-free TNA extraction (Fig. 4B,C). As RNA constitutes a small portion of the nucleic acids present in an extraction, the majority of the reads can be assigned to DNA; these are identifiable as spanning intron–exon boundaries. To identify RNA derived reads, cDNA contigs were used for mapping and doing so allowed for clear identification of intron spanning reads indicating successful capture of cfRNA.

## Discussion

In recent years, many library preparation approaches have been adapted to testing of clinical material isolated from liquid biopsies, and they have improved the specificity of the data produced by reducing the background error rate of these approaches^8–13^. However, current technologies have inherent limitations that arise from the nature of PCR and ligation-based approaches. Because of this, new library preparation approaches that are better able to assess the full spectrum of nucleic acids present in a liquid biopsy sample—including single stranded, short, and damaged DNA and/or RNA molecules—could lead to significant improvements to clinical testing.

In this paper we have described a new technology which is uniquely suited for efficient and rapid capturing of nucleic acids extracted from a liquid biopsy; **A**daptor **T**emplate **O**ligo **M**ediated **Seq**uencing—ATOM-Seq. This technology is a highly robust, flexible and rapid approach for the generation of NGS libraries using DNA, RNA, or DNA + RNA mixtures as starting material. We have demonstrated its high sensitivity and specificity for detection of clinically relevant mutations (including SNVs, deletions/insertions) from reference and clinical material, showing excellent performance with both FFPE DNA and cfDNA samples. This technology is based on oligo annealing and polymerase driven extensions and amplifications, which form the basis of all PCR reactions and therefore ATOM-Seq inherits the same natural high efficiency and efficacy. The nature of the ATOM-Seq technology and its uniquely designed protocol imparts numerous advantages. These are all founded on its ability to capture the free 3′ ends of any available nucleic acid, including double- and single-strand DNA, enzymatically fragmented or sonicated FFPE-derived DNA, cfDNA, and single- or double-strand cDNA derived from total RNA (high quality or FFPE), cfRNA or cfDNA + cfRNA from a total cell-free nucleic acid extraction. As any free 3′ end can be captured, the technology will also be resistant to highly damaged and fragmented DNA. Combined, these factors mean ATOM-Seq is capable of capturing a much greater breadth of starting material when compared to other commercially adopted PCR and ligation-based approach. This makes ATOM-Seq the ideal technology for use with clinical samples, especially liquid biopsy samples. Notably however ATOM-Seq does suffer reduced efficiency when using DNA that has been mechanically sheared by methods such as sonication. This is because a proportion of molecules prepared in this manner lack the free 3′ groups on original molecules required for effective use as a primer during the ATO reaction. This is also true, but to a lesser extent, with ligation based approaches as they typically include ‘end repair’ steps which will help to re-expose some of the blocked 3′ ends making them available for ligation. Fortunately, this can be entirely avoided by the use of enzymatic fragmentation approaches, such as those included with library preparation approaches from Qiagen and ArcherDX.

ATOM-Seq’s unique advantages are also present in the workflow after the initial DNA capture. The most substantial of these is the linear amplification which generates multiple copies of each original molecule. This is a central part of the protocol for two reasons: firstly, it allows for greater sensitivity and flexibility by enabling samples to be divided between multiple downstream PCR reactions without reducing sensitivity, as this amplification allows representation of all starting material in every reaction. Without this prior amplification, splitting a sample would incur an immediate 50% drop in maximum sensitivity. This sample division also enables the use of multiple primer pools for independent targeting of both the sense and antisense DNA strands. This in turn allows for simpler dual strand whole exon coverage of tumour suppressors such as *TP53* and *APC* by avoiding the risk of opposing primers forming non-specific PCR products while allowing for reductions in PCR cycles from increased amplification efficiencies. These impart advantages over PCR-based approaches which inevitably struggle due to limitations with tiling primers^8^. However, using ATOM-Seq to develop very large panels such as those targeting whole exomes offered by hybridisation-based technologies will require extra effort to design.

The second advantage of incorporating a linear amplification is that it aids in the reduction of the error rate in the final library by improving the reliability and power of UMIs. In approaches where UMI incorporation is followed immediately by PCR amplification, any polymerase errors that were to occur in the first round of target PCR would propagate exponentially and form a substantial fraction of the final PCR products^20^. The rounds of linear amplification in the ATOM-Seq protocols mean any randomly generated errors would be present in a far smaller fraction of final PCR products, reducing the chance that these are still present following error correction. Having the genetic information for both sense and antisense strands independently also allows for the comparison of mutations between strands, which allows for increased confidence in mutation calling.

The presence of UMIs in ATOM-Seq’s protocol allows for their use for enhanced error correction capabilities, but due to their random lengths there may be situations where this feature adds additional complexities to the NGS data analysis. For example, it is not possible to perfectly trim the UMI from reads prior to their mapping. This in turn may lead to situations where mapping software could identify a SNP/insertion/deletion due to random similarities between a long UMI and reference sequences rather than not mapping the UMI. Additionally for ‘fragmentomics’, which is the study of break points in cell-free DNA, the UMI lengths may add additional levels of correction necessary to infer the break point positions. Though as we have mapped the length distribution this information could be used to filter the data to obtain accurate breakpoint distributions.

Current technologies have not, or cannot, incorporate this linear amplification and sample division method. PCR-based protocols incorporate UMIs in the first few rounds of PCR^8^. As such any attempt to incorporate a linear amplification would result in generation of errors which could not be differentiated from real mutations, defeating the purpose of UMI incorporation entirely. Technologies which combine ligation and PCR such as Anchored Multiplex PCR or Single Primer Extension^11–13^, apart from Qiagen’s GeneRead QIAact AIT DNA UMI Panel and an early published version of the AMP technology^11^, do not divide their samples for independent strand targeting. As their universal primer sites are on the 5′ ends of DNA, not the 3′ as in ATOM-Seq, this means that linear amplification would have to be performed using their pools of targeted specific primers. Primer pools containing opposing pairs of primers would, (1) likely introduce bias due to variation in primer efficiencies and (2) inevitably produce PCR products, at the detriment of the protocol. Ligation + capture-based protocols could support linear amplification as they create 3′ universal primer sites, but doing this would add complexity to protocols which can already be relatively long and complex.

The aforementioned advantages of ATOM-Seq are supported through to the performance of the technology with the different types of reference and clinical materials. The presented data demonstrate 100% detection of ≥ 5.0% AF mutations with as little as 0.5 ng of reference material and with between 10 and 40 ng of sonicated and enzymatically fragmented FFPE material. Not only does ATOM-Seq perform very well with this material, but when the identified mutations from clinical samples were compared with the allele frequencies determined by other technologies (pyrosequencing, ligation + PCR NGS, or PCR NGS) there was a very strong correlation between these data. This demonstrates that despite ATOM-Seq being a relatively young and unique technology it performs very well when compared with other much more mature technologies. Though ATOM-Seq was developed with liquid biopsy testing in mind, it is important to note that it also performs very well with FFPE material; formalin fixation still forms a central step in oncology patient sample processing worldwide.

We also demonstrate achieving up to 100% detection of 1% AF mutations with 1–5 ng of material and 94% detection of ≥ 0.1% AF mutations with 20 ng of material when testing reference samples fragmented to be representative of cfDNA’s size distribution. Using 5 ng of reference material we identified a list of inferred ground truth mutations, a combination of those known to be present (i.e. those certified by the manufacturer) and those we identified as shared between replicate samples, which demonstrated sensitivities and specificities of 99.2% and 98.3% respectively. This dropped slightly to 97.9% and 97.3% respectively when only looking at very low frequency (≤ 2% allele frequency) mutations. A false positive rate of ~ 23–30 per megabase was also identified, which we expect would improve in the future by having a bespoke variant caller trained on the ATOM-Seq technology to more reliably filter out false positives and identify true positives. We do note that we were only able to infer a ‘ground truth’ as a base line for those experiments; follow up studies which use highly validated genome in a bottle samples (along with an aforementioned trained variant caller) would help to further confirm the specificity of this technology.

While using liquid biopsy extracted material, we were able to identify concordant mutations down to 0.12% AF in cfDNA, and in this pilot study generated a high level of concordance between paired FFPE and cfDNA samples, 15 of 18 paired samples. In a limited sample set (n = 3) ATOM-Seq also outperformed Qiagen’s ligation-based NGS technology by detecting more cfDNA mutations: 8 versus 2. Due to the limited comparison a larger cohort is needed to confirm this advantage such that variables including sample variability can be more accurately controlled for. However, given the equivalent molecular depths between technologies at some of these sites the improved performance could in part be attributed to ATOM-Seq’s ability to capture a greater proportion of cfDNA which is enriched for tumour derived material (short and single stranded DNA fragments; Supplementary Table 9). ATOM-Seq was also able to identify all expected types of mutations from paired FFPE and cfDNA clinical material, including SNPs, deletions as long as 28 bp (found in *BRCA2*) and insertions up to 9 bp (found in *ERBB2;* Supplementary Table 9).

Not only do ATOM-Seq-based approaches contain many inherent advantages, but targeted enrichment from cfDNA to purified final library can be accomplished in only 5.75 h, a 1-day protocol (Fig. 1). When using RNA, proceeding from RNA/cfRNA/TNA to a bead purified final library can be completed in just over 7 h (Supplementary Fig. 1). ATOM-Seq protocols are more time efficient when compared to ligation based protocols, which can take from eight hours for ligation + PCR protocols to up to three days for ligation + capture protocols^8–13^.

ATOM-Seq’s excellent performance with both FFPE material and especially with cfDNA demonstrates that it would be an ideal technology for incorporation into both research and clinical oncology sample testing environments. The combination of the underlying approach, streamlined flexible protocols, and technological advantages establishes ATOM-Seq as perfectly and uniquely suited for adoption into testing for cancer associated mutations using challenging liquid biopsy material.

## Methods

### Preparation of starting materials

Reference material was purchased from Horizon Discovery, Tru-Q 4 (HD731) containing 5% allele frequency mutations, Tru-Q 7 (HD734) containing 1.3% allele frequency mutations, and a wild type (WT) sample Tru-Q 0 (HD752). In order to create a 0.13% allele frequency sample, the 1.3% material was diluted 1:10 in the WT material. To create a fragmented DNA analogue with a size distribution similar to that of cfDNA, the 5%, 1.3%, and 0.13% samples were independently fragmented using the KAPA Frag kit (Roche; 07962517001) following the manufacturers recommended conditions followed by a 3 × AMPure XP bead purification. FFPE samples were either enzymatically fragmented as above or sonicated using a Covaris ultrasonicator. The size distribution was measured using an Agilent Bioanalyzer High Sensitivity kit and concentrations were determined using a Qubit dsDNA HS Assay Kit (Thermo Fisher Scientific; Q32854).

Fusion reference material was purchased from Horizon Discovery, an FFPE sample containing 3 validated fusions (HD784). RNA was extracted using a ReliaPrep FFPE Total RNA Miniprep System kit (Promega; Z1001) following the manufacturer's recommended protocol. Frozen serum was purchased Cambridge Bioscience Ltd (PLSSKF8BC200-XSXX) and total nucleic acids were extracted using MagMAX Cell-Free Total Nucleic Acid Isolation Kit following the manufactures recommended conditions (Applied Biosystems; A36716).

### Primer panel design

A pan cancer hotspot panel targeting the most frequently mutated sites across a broad range of cancers was designed by examining COSMIC database version 85. The pan caner panel was used while testing specificities and sensitivities on reference materials. The primer sequences were designed taking into account coverage of target sites, balancing primer Tm, and predicted specificity within the human genome (hg38). The final full pan cancer panel includes 100 genes which cover approximately 4000 annotated COSMIC mutations. There are 577 primer sites targeting the sense DNA strand and 570 primer sites targeting the antisense DNA strand for a total panel size of 59 kb. All enrichment amplifications are designed to have two rounds of PCR, and as such an ‘outer’ and ‘inner’ primer were designed for all target sites, where the inner primers contain a universal tail to allow for preparation of dual index libraries. All primers were synthesised by Eurofins Genomics. Two smaller 22 or 23 gene panels were created as a subset of the pan cancer panel for specifically testing colon cancer and lung cancer cfDNA and FFPE clinical samples. The smaller panels have 146 or 180 primers targeting the sense DNA strand and 151 or 181 targeting the antisense DNA strand. Fusion panel design followed an equivalent process as the pan cancer panel except for targeting gene fusions frequently detected in cancer. Placement of enrichment primers in a conserved fusion partner allows for detection of unknown fusion partners. The fusion panel targeted 169 conserved exons found as fusion partners across 22 genes. Details of all target genes are in Supplementary Table 11.

### ATO reaction: 3′ extension reaction

For reference material testing, different quantities of fragmented DNA were used in triplicate for each allele frequency being tested. For 5% allele frequency either 0.5 ng or 1.0 ng was used, for 1.3% either 1.0 ng or 5.0 ng was used, and for 0.13% 20 ng was used. When testing fusion detection with reference material, a total of 100 ng of RNA was used. For TNA a total of 13.65 ng was used as determined by DNA mass, measured using a Qubit 3.0. For clinical material various quantities were used. For the first pilot study 10 ng of Covaris-sheared FFPE-extracted DNA were used. For the second pilot study 40 ng of enzymatically fragmented FFPE and between 11.8 and 20.96 ng of cfDNA were used. For the third pilot study 40 ng of enzymatically fragmented FFPE DNA and between 25 and 64 ng of cfDNA were used.

All methods (depicted in Fig. 1A, Supplementary Figs. 1, 2) were performed with development pre-release versions of the XCeloSeq Targeted DNA Enrichment, XCeloSeq Targeted RNA Enrichment, or XCeloSeq cfDNA Library Preparation protocols.

When RNA was used, first strand cDNA was made using SuperScript IV First-Strand Synthesis System (ThermoFisher, 18091050) with total RNA or total cell-free nucleic acids. The sample was thermocycled as follows: 65 °C for 5 min, 4 °C for 2 min, 25 °C for 2 min, and 55 °C for 10 min. To this 1.5 µl of an equal mix of DNA Polymerase I and RNase H (NEB M0209S, M0297S) was added to the first strand cDNA and incubated at 22 °C for 30 min. Second strand cDNA was purified with AMPure XP beads with a 2.0 × ratio and eluted into 13 µl of nuclease free water.

The following steps vary slightly depending on the samples and version of the protocol used. All samples followed the same general procedure. The samples, be they previously purified double strand cDNA or cDNA/cfDNA mixture, cfDNA, fragmented FFPE DNA, or ssDNA oligos, were either combined with 2 µl of ATO to a final volume of 15 µl for targeted RNA or DNA enrichment protocols, or were combined with 1 µl of ATO to a final volume of 7.5 µl for the whole sample enrichment protocols. This mixture was heated to 65 °C for 2.5 min before being cooled to 4 °C. To this either 2 µl of Phi29 buffer, 1 µl of 10 mM dNTPs, 1 µl of Phi29 and 1 µl of Bsu DNA polymerase (ThermoFisher, EP0092; NEB, N0447L, M0330L) were added to targeted RNA or targeted DNA enrichment protocols or half the equivalent volumes were added to whole sample enrichment protocols for a final volume of 20 µl or 10 µl. To induce the extension of the starting material as a primer using the ATO as a template the mixture is placed in a pre-cooled 4 °C thermocycler and cycled as follows, 26 °C for 6 min, 30 °C for 10 min, 65 °C for 1 min, 10 °C for 1 min, 26 °C for 6 min, 30 °C for 10 min followed by two cycles of 65 °C for 1 min, 10 °C for 1 min, 26 °C for 6 min and 30 °C for 5 min. For targeted DNA and RNA enrichment protocols 2 µl, and for whole sample protocols 1 µl, of USER enzyme (NEB, M5505L) was added and incubated for 37 °C for 20 min and 25 °C for 10 min. For targeted RNA and targeted DNA enrichment protocols 1 µl of 50 µM linear amplification primer (5′-GTGACTGGAGTTCAGACGTGTGCTCTTCCG*A-3′) and 24 µl of Phusion Hot Start Flex 2X Master Mix (NEB, M0536L) were combined and cycled as follows, 98 °C for 30 s followed by 10 cycles of 98 °C for 5 s, 60 °C for 1 min, and 72 °C for 1 min and with a final extension of 72 °C for 2 min. For whole sample library protocols 1.5 µl of the linear amplification primer and 12.5 µl of Phusion Hot Start Flex 2X Master Mix were combined and cycled as follows 98 °C for 30 s followed by 6 cycles of 98 °C for 10 s, 65 °C for 75 s, and with a final extension of 65 °C for 2 min. The product of this linear amplification is the ‘First Amplification Product’ which is used for all downstream steps.

### ATOM-Seq target hotspot enrichment

For the first PCR in the targeted DNA enrichment protocol, 25 µl of Phusion Hot Start Flex 2X Master Mix, 0.5 µl linear amplification primer, 2.5 µl of a primer pool and 22 µl of the First Amplification Product were combined. Two such reactions are prepared per sample, using primer pools that targeted either the sense or antisense DNA strands, respectively. Both of these mixtures were thermocycled as follows, 98 °C for 30 s followed by 14 cycles of 98 °C for 5 s, 65 °C for 5 min, and 72 °C for 30 s followed by a final extension of 72 °C for 2 min. These Second Amplification Products were purified with AMPure XP beads with a 1.8 × ratio. For the second PCR, 25 µl Phusion Hot Start Flex 2X Master Mix, 2.0 µl of a nested primer pool targeting the sense or the antisense DNA strand, 1.0 µl of an i7 index primer and 1.0 µl of an i5 index primer and 21 µl of the appropriate bead purified first PCR product were combined. Both of these mixtures were thermocycled as previously. Finally, both samples were purified with AMPure XP beads with a 1.2 × ratio.

### ATOM-Seq targeted fusion enrichment

For the targeted RNA enrichment protocol the first PCR differs in that there is only a single pool of primers. As such, 0.5 µl linear amplification primer, 2.0 µl of a primer pool targeting conserved fusion exons and an additional 1.5 µl of Phusion Hot Start Flex 2X Master Mix were added directly to the entire 46 µl First Amplification Product, which was then cycled as previously. These were also bead purified and amplified again following the procedure for the targeted DNA enrichment protocol using nested primers targeting conserved fusion exons. A schematic of the protocol is detailed in Supplementary Fig. 1.

### ATOM-Seq whole sample protocol

When ssDNA oligos were used as input, a normal ATO Reaction, first amplification and 2 × AMPure XP bead purification step were performed as detailed above, producing molecules with Illumina-compatible 5′ universal tails. Note, some input oligos already possessed this 5′ tail and so these initial steps were skipped for those oligos.

Following this a second ATO Reaction was preformed using either the First Amplification product or pre-tailed ssDNA oligo as a template. The steps are equivalent to the first ATO Reaction (including USER treatment) except an ATO with a different universal priming site was used with cycling as follows: 26 °C for 6 min, 30 °C for 10 min, 65 °C for 1 min, 10 °C for 1 min, 26 °C for 6 min, 30 °C for 10 min.

Finally a global sample amplification using the 22 µl of the USER treated second ATO Reaction, 1.5 µl of an i5 index primer and 1.5 µl of an i7 index primer and 25.0 µl of Phusion Hot Start Flex 2X Master Mix were combined and used to produce a final sequencing library cycled as follows 98 °C for 30 s, 8 cycles of 98 °C for 10 s 60 °C for 30 s, 65 °C for 75 s followed by a final incubation of 65 °C for 2 min. These libraries were purified by a 0.9 × and a 0.7 × AMPure XP bead purification.

### Sequencing and data analysis

Sequencing was generated using either a MiSeq or NextSeq. High depth sequencing was generated using a HiSeq at the Wellcome Centre for Human Genetics, Oxford. Reference material sequencing data was generated using a MiSeq. For the first pilot study each sample was sequenced to a depth between 0.7 and 5.1 million 150 bp PE reads on a MiSeq. For the second pilot study each FFPE and cfDNA sample was sequenced with between 4.6 and 5.6 million 150 bp PE reads on a single NextSeq run. For the third pilot study each FFPE sample was sequenced with between 3.3 and 79 million 150 bp PE reads and each cfDNA sample between 2.9 and 79 million 150 bp PE reads on either a MiSeq or HiSeq.

Sequencing data was processed using the following steps. Initially, adaptors were trimmed from reads, 20 bp UMIs were copied from the beginning of read 2 into read headers (required for gencore), and inserts below 30 bp were discarded using fastp v0.20.1^21^ with default settings alongside custom python scripts. The trimmed reads were then mapped to the hg38 reference genome using BWA v0.7.15^22^ with default settings. On target rate was measured as the percentage of reads which mapped to expected PCR primer sites relative to the total number of mapped reads. Read uniformity was determined as the percentage of reads which had a read depth greater than 20% of the mean read depth. Mapped reads were passed to gencore v0.15.0^23^ for consensus read generation and error correction using default settings (80% consensus to generate a consensus base) with a minimum family size of two. Consensus reads were parsed by a custom python script which removed any reads not mapping to a primer site and trimmed the primer sequence from those reads which did map to a primer site to remove them from variant calling. Variants were then called by passing the filtered and trimmed consensus reads to VarDict v1.7^24^ and calling variants only within the targeted regions using the following settings -f 0.0001 -r 1 -M 10 -P 0 -Q 10 -U -u. Variant lists were then filtered further to remove variants with AF < 5.0% and counts < 3 from FFPE samples along with additional manually inspecting consensus reads in Integrative genomics Viewer^25^. Variance of reference material mutations was determined for all mutations individually, and, of the averages across all mutations. The variation was calculated by determining the sum of all the squared differences from the mean divided by the number of data points. Average allele frequencies and calculated variances are shown in Supplementary Tables 3–5.

To assess the length distribution of the UMIs generated during an ATO reaction, a pool of PCR primers was used as starting material in combination with the above described whole sample protocol. Sequencing reads were processed as above. The on-target reads were further trimmed to remove the input primer sequence and determine the length of the 3′ UMI. The frequency and distribution all UMI lengths were summed their frequency and distribution (Fig. 1, Supplementary Table 1). To determine the effective UMI complexity the frequency of each UMI length was normalised to the frequency of the shortest UMI, whose complexity is expected to saturate the quickest. The 8 bp UMI, made of a stretch of 5Ns, has a theoretical maximum complexity of 1024 (4^5^), as such the frequency of all other UMI were determined relative to this number. This was done to estimate the number of identical DNA molecules which could be captured before you will have duplicate UMIs.

To assess the specificity of the ATOM-Seq protocol we approximated the ‘inferred ground truth’ from three reference material replicates. This was done by generating a combined list of variants across the three replicates generated using the 1% AF with 5 ng of starting material. A minimum variant count of 3 was used as a cut off, any variants below this threshold were treated as errors and discarded. From this list ‘inferred true positives’ were assessed to be those which were present in 2 or 3 of the 3 replicates (Supplementary Fig. 3).

All sequencing data generated using RNA or TNA was processed with the following pipeline. Raw reads were trimmed as before, mapped to the hg38 reference genome using the splice aware mapper STAR^26^ or to cDNA contigs using BWA. Mapped reads were used to identify expected reference fusions as well as to geerate on-target counts. Custom fusion contigs were used to accurately depict fusion spanning reads [COSF408 (EML4-ALK); COSF1271 (CCDC6-RET); COSF1196 (SLC34A2-ROS1)]. For cell-free total nucleic acids, cfDNA derived reads were identified based on the reads spanning intron/exon boundaries, cfRNA derived reads were identified being exon junction spanning after mapping to JAK1 mRNA contigs (NM_001320923.1).

## Supplementary Information

Supplementary Information.

## Data Availability

The datasets generated in the current study are available from the corresponding author upon reasonable request.
