# Supplementary material for: Adaptor Template Oligo-Mediated Sequencing (ATOM-Seq) is a new ultra-sensitive UMI-based NGS library preparation technology for use with cfDNA and cfRNA
Source: Sci Rep. 2021 Feb 4;11:3138. doi: 10.1038/s41598-021-82737-9 (PMC7862664; doi:10.1038/s41598-021-82737-9)
Supplement: Supplementary file 1 — Supplementary Information. [file 41598_2021_82737_MOESM1_ESM.pdf]

Adaptor Template Oligo-Mediated Sequencing (ATOM-Seq) is a new ultra-sensitive UMI-based NGS library preparation technology for use with cfDNA and cfRNA

Thomas L. Dunwell, Simon C. Dailey, Anine L. Ottestad, Jihang Yu, Philipp W. Becker, Sarah Scaife, Susan D. Richman, Henry M. Wood, Hayley Slaney, Daniel Bottomley, Xiangsheng Yang, Hui Xiao, Sissel G. F. Wahl, Bjørn H. Grønberg, Hongyan Dai, Guoliang Fu.

Supplementary Information

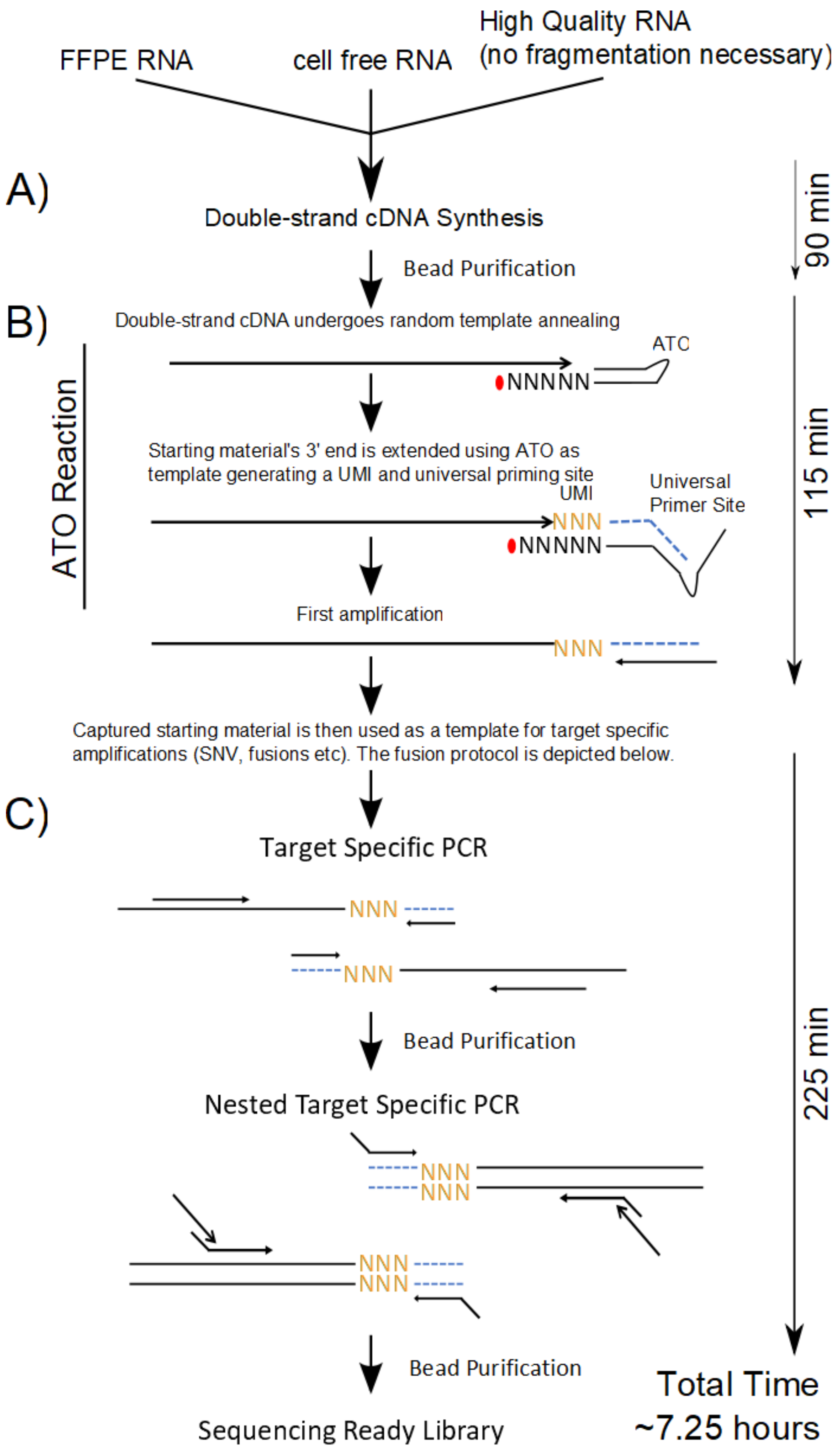

Supplementary Figure 1. Overview of an ATOM-Seq Fusion enrichment protocol. **A)** The first step is double strand cDNA synthesis followed by a bead purification. **B)** An "ATO Reaction" then captures the ds-cDNA and 3' ends are extended by a polymerase, following which is an initial round of linear amplification. **C)** The whole of the initial linear amplification is used as template in two rounds of nested PCR using target specific enrichment primers.

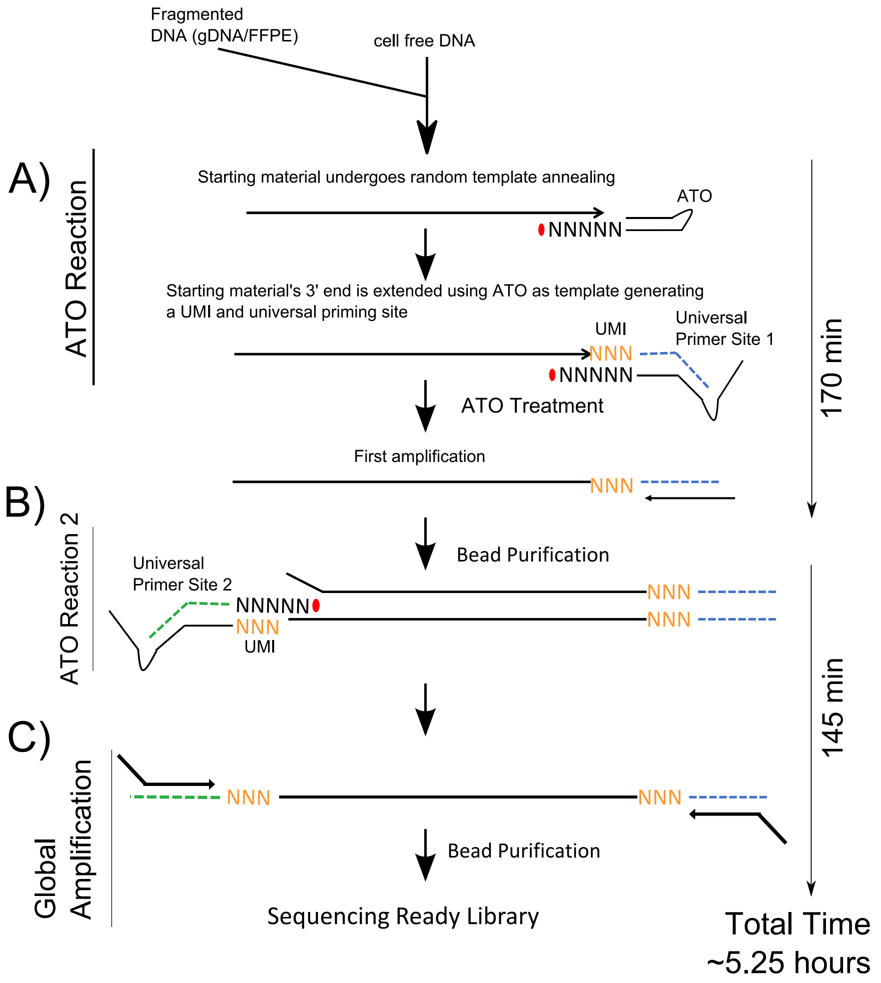

Supplementary Figure 2. Overview of an ATOM-Seq 'whole sample' protocol. ATOM-Seq based protocols can take any suitable stating material including cell-free DNA, fragmented FFPE DNA, or cDNA. **A)** The first step is an "ATO Reaction" which captures the starting material by annealing a synthetic Adaptor Template Oligo (ATO) to the 3' ends of all starting material. The 3' ends are then extended by a polymerase which uses the ATO as a template. This extension generates a Unique Molecular Identifier and a universal primer site on all 3' ends, following which is the first amplification. **B)** The first amplification product is used as an input for a second ATO-Reaction which creates a second, differing universal primer binding site. **C)** The two universal primer binding sites are used for whole library amplification.

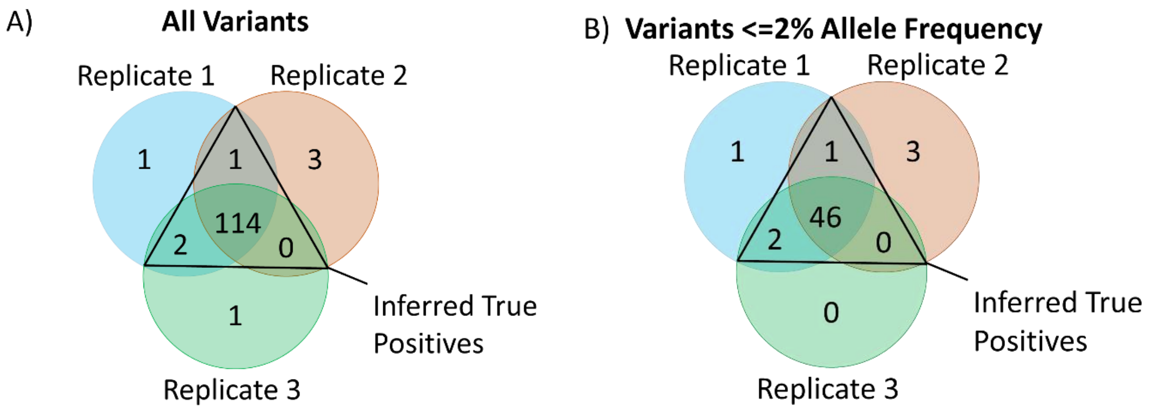

Supplementary Figure 3. Number of variants detect in each of three biological replicates with a variant count greater than 3. The variants are separated into those detected in only one sample, in 2 of 3 samples, or in all 3 samples. Those samples present in 2 or 3 of 3 samples were deemed ‘inferred true positives’ and those variants in only 1 sample were ‘inferred false positives’ **A)** Indicates all variants detected across the 3 biological replicates irrespective of allele frequency and **B)** indicates those variants with an AF equal to or below an average allele frequency of 2%.

UMI Length	Theoretical Complexity of Each UMI Length	% of reads with each length UMI	Actual contributed complexity of each UMI length	Saturation of each UMI length
8	1E+03	12%	1024	100.00%
9	4E+03	15%	1308	31.92%
10	2E+04	14%	1217	7.43%
11	7E+04	13%	1071	1.63%
12	3E+05	11%	974	0.37%
13	1E+06	10%	845	0.08%
14	4E+06	8%	720	0.02%
15	2E+07	6%	553	0.00%
16	7E+07	5%	417	0.00%
17	3E+08	3%	238	0.00%
18	1E+09	1%	97	0.00%
19	4E+09	0%	25	0.00%
20	2E+10	0%	13	0.00%
21	7E+10	0%	8	0.00%
22	3E+11	0%	7	0.00%
23	1E+12	0%	5	0.00%
24	4E+12	0%	4	0.00%
Theoretical Max Complexity	6E+12	Total Effective Complexity	8525	

Supplementary Table 1. UMI length distribution and frequency counts. UMI lengths begin at 8bp, which corresponds to length of the protected barcode. The percentage of reads of each UMI length was determined using DNA oligos of known length and sequence. The contribution of each UMI length to the total complexity of all UMIs was determine by normalising their relative depth to the UMI which would saturate first, the 8bp UMI.

Allele Frequency	Input DNA Mass (ng)	Sequencing Instrument	PE Reads	PE Reads after trimming	Genome Mapping Rate (%)	On-Target Reads	Total Discarded Reads (%)	Average Molecular Depth Across All Primers	Average UMI Family Duplication Rate
5.00%	0.5	MiSeq	560,472	524,581	99.72	457,309	18.41	40	10
5.00%	0.5	MiSeq	495,204	482,520	99.57	422,084	14.77	41	9
5.00%	0.5	MiSeq	613,403	603,404	99.59	531,484	13.35	44	11
5.00%	1	MiSeq	1,192,952	1,141,461	99.84	1,004,301	15.81	85	10
5.00%	1	MiSeq	1,162,252	1,134,866	99.80	1,004,050	13.61	86	10
5.00%	1	MiSeq	1,200,695	1,190,049	99.75	1,052,164	12.37	89	10
1.30%	1	MiSeq	1,237,906	1,195,681	99.76	1,033,082	16.55	92	10
1.30%	1	MiSeq	1,037,135	1,022,806	99.72	892,801	13.92	89	9
1.30%	1	MiSeq	1,732,498	1,717,585	99.76	1,541,547	11.02	105	13
1.30%	5	MiSeq	3,076,461	3,064,987	99.76	2,692,278	12.49	331	7
1.30%	5	MiSeq	3,420,025	3,402,241	99.76	2,923,725	14.51	348	7
1.30%	5	MiSeq	2,727,389	2,690,581	99.81	2,365,084	13.28	306	7
0.13%	20	MiSeq	13,040,066	12,985,790	99.76	11,287,697	13.44	1112	9
0.13%	20	MiSeq	13,230,186	13,161,508	99.77	11,331,558	14.35	1124	9
0.13%	20	MiSeq	11,714,553	11,673,354	99.82	10,271,511	12.32	1067	8

Supplementary Table 2. Details of sequencing for DNA reference standards.

						0.5 ng Reference Material		1.0 ng Reference Material	
Chromosome	Base Pair	Reference	Mutation	Gene	Expected Allele Frequency	Detect Allele Frequency (Average)	Detect Allele Frequency Variance	Detect Allele Frequency (Average)	Detect Allele Frequency Variance
chr9	130872896	C	T	ABL1	0.05	0.086	0.000988839	0.060	0.00012
chr7	140753336	A	C	BRAF	0.04	0.038	0.000232194	0.035	0.00025
chr7	55191831	T	A	EGFR	0.042	0.057	6.45872E-05	0.060	0.00064
chr15	90088606	G	A	IDH2	0.05	0.068	8.01106E-05	0.065	0.00026
chr4	54733155	A	T	KIT	0.05	0.070	0.000340313	0.061	0.00013
chr12	25245350	C	T	KRAS	0.05	0.050	0.001279471	0.033	5.5E-05
chr12	25245351	C	A	KRAS	0.05	0.063	0.000641847	0.036	0.00011
chr12	25227341	A	C	KRAS	0.05	0.048	0.000131269	0.049	1.3E-05
chr1	114713908	A	G	NRAS	0.05	0.075	0.000521862	0.074	2.7E-05
chr4	54285926	A	T	PDGFRA	0.05	0.023	0.000259572	0.040	6.3E-07
					Min	0.02		0.03	
					Max	0.09		0.07	
					Average	0.06		0.05	
					Variance of all Allele Frequency Average	0.00031587		0.000196	

Supplementary Table 3. Detected and expected allele frequencies for 5% reference mutations with 0.5 ng and 1.0 ng of reference material. Individual mutation averages and variances are of 3 biological replicates.

						1.0 ng Reference Material		5.0 ng Reference Material	
Chromosome	Base Pair	Reference	Mutation	Gene	Expected Allele Frequency (%)	Detect Allele Frequency (Average, %)	Detect Allele Frequency Variance	Detect Allele Frequency (Average, %)	Detect Allele Frequency Variance
chr9	130872896	C	T	ABL1	1.30%	1.65	1.8E-05	1.41	1.68889E-05
chr2	29220829	G	T	ALK	1.30%	1.69	1.7E-04	0.53	3.71894E-05
chr7	140753336	T	G	BRAF	1.00%	2.12	0.00015	0.88	1.87E-06
chr7	140753336	GT	AA	BRAF	1.00%	0.92	1.58E-05	0.97	8.61E-06
chr7	140753337	G	A	BRAF	1.00%	0.59	1.74E-05	1.06	6.72E-07
chr7	140753336	GT	AG	BRAF	1.00%	0.79	3.54E-05	0.96	7.39E-07
chr7	55191822	T	G	EGFR	1.00%	1.24	9.8E-05	0.96	1.18002E-06
chr7	55191831	T	A	EGFR	1.00%	0.59	7.0E-05	0.88	9.94667E-06
chr7	55181378	C	T	EGFR	1.00%	0.31	6.0E-06	1.26	1.07785E-05
chr7	55174771	ELREA	-	EGFR	1.00%	0.69	8.7E-06	0.84	5.35722E-06
chr10	121520163	C	ls	FGFR2	1.00%	1.16	1.3E-05	1.00	3.60722E-06
chr13	28018505	G	T	FLT3	1.30%	1.13	9.8E-06	1.04	1.04417E-06
chr13	28018497	I	-	FLT3	1.30%	1.37	2.5E-05	0.94	7.67722E-06
chr19	3118944	A	T	GNA11	1.30%	1.85	4.5E-06	1.23	6.19691E-06
chr9	77794572	T	A	GNAQ	1.30%	1.19	4.0E-05	1.14	5.33045E-06
chr2	208248388	C	T	IDH1	1.30%	1.01	2.4E-05	1.06	4.97549E-06
chr2	208248389	G	A	IDH1	1.30%	0.44	1.4E-06	0.80	3.62336E-06
chr15	90088702	G	A	IDH2	1.30%	2.17	4.8E-05	0.82	1.5522E-06
chr15	90088606	G	A	IDH2	1.30%	1.10	9.6E-05	1.37	2.03355E-05
chr9	5073770	G	T	JAK2	1.30%	0.86	5.1E-05	1.06	1.40581E-05
chr4	54733155	A	T	KIT	1.30%	0.13	3.6E-06	0.90	8.43469E-06
chr12	25225628	G	A	KRAS	1.30%	1.20	6.4E-05	1.01	4.09412E-06
chr12	25245350	G	C	KRAS	1.30%	0.69	9.9E-06	1.13	2.85721E-06
chr12	25245350	G	T	KRAS	1.30%	1.32	4.6E-05	0.87	3.03839E-07
chr12	25245350	G	A	KRAS	1.30%	0.57	1.7E-05	1.12	2.80117E-06
chr12	25245351	G	C	KRAS	1.30%	0.65	2.2E-05	0.92	1.02389E-06
chr12	25245351	G	A	KRAS	1.30%	1.59	2.6E-05	1.35	9.66776E-06

chr12	25245351	G	T	KRAS	1.30%	0.83	5.3E-05	0.79	6.35476E-06
chr12	25227341	A	C	KRAS	1.30%	0.94	3.2E-05	0.83	4.99272E-07
chr12	25227342	A	T	KRAS	1.30%	1.46	3.9E-05	0.94	5.07707E-06
chr15	66436825	C	T	MEK1	1.30%	1.01	3.9E-06	0.93	6.07476E-06
chr7	116783374	T	G	MET	1.00%	0.34	4.2E-07	0.80	4.58527E-06
chr9	136504892	A	G	NOTCH	1.30%	0.95	5.0E-05	0.91	4.81845E-05
chr1	114713907	A	T	NRAS	1.30%	1.61	4.5E-05	1.02	8.60195E-06
chr1	114713909	C	A	NRAS	1.30%	1.80	6.4E-06	1.68	2.22056E-06
chr1	114713908	A	T	NRAS	1.30%	0.79	5.7E-05	1.03	6.08317E-06
chr1	114713908	A	G	NRAS	1.30%	1.78	8.8E-05	1.10	2.33217E-07
chr4	54285926	A	T	PDGFRA	1.30%	1.66	5.1E-05	1.85	6.97606E-05
chr3	179218294	G	A	PIK3CA	1.30%	0.70	9.8E-05	0.32	1.42969E-06
chr3	179218303	G	A	PIK3CA	1.30%	0.61	1.3E-06	0.64	8.02606E-07
					Min	0.13		0.32	
					Max	2.31		1.85	
					Average	1.09		1.01	
					Variance of all Allele Frequency Average	0.265271972		0.073181936	

Supplementary Table 4. Detected and expected allele frequencies for 1.3% reference mutations with 1.0 ng and 5.0 ng of reference material. Individual mutation averages and variances are of 3 biological replicates.

						20 ng Reference Material	
Chromosome	Base Pair	Reference	Mutation	Gene	Expected Allele Frequency (%)	Detect Allele Frequency (Average, %)	Detect Allele Frequency Variance
chr9	130872896	C	T	ABL1	0.13%	0.13	2.76872E-07
chr2	29220829	G	T	ALK	0.13%	0.04	2.592E-07
chr7	140753336	T	G	BRAF	0.10%	0.12	8.91807E-08
chr7	140753336	GT	AA	BRAF	0.10%	0.13	8.49239E-07
chr7	140753337	G	A	BRAF	0.10%	0.12	2.17413E-07
chr7	140753336	GT	AG	BRAF	0.10%	0.03	1.88074E-07
chr7	55191822	T	G	EGFR	0.10%	0.11	1.35874E-06
chr7	55191831	T	A	EGFR	0.10%	0.13	3.7745E-07
chr7	55181378	C	T	EGFR	0.10%	0.09	2.67159E-07
chr7	55174771	ELREA	-	EGFR	0.10%	0.08	3.13348E-07
chr10	121520163	C	ls	FGFR2	0.10%	0.26	2.51208E-06
chr13	28018505	G	T	FLT3	0.13%	0.14	1.08075E-07
chr13	28018497	I	-	FLT3	0.13%	0.18	4.98503E-08
chr19	3118944	A	T	GNA11	0.13%	0.14	3.99106E-07
chr9	77794572	T	A	GNAQ	0.13%	0.08	3.50828E-07
chr2	208248388	C	T	IDH1	0.13%	0.09	2.10526E-07
chr2	208248389	G	A	IDH1	0.13%	0.17	7.95022E-07
chr15	90088702	G	A	IDH2	0.13%	0.15	9.3182E-07
chr15	90088606	G	A	IDH2	0.13%	0.07	8.88889E-07
chr9	5073770	G	T	JAK2	0.13%	0.11	3.59601E-07
chr4	54733155	A	T	KIT	0.13%	0.15	1.0706E-06
chr12	25225628	G	A	KRAS	0.13%	0.18	7.17929E-07
chr12	25245350	G	C	KRAS	0.13%	0.08	1.55673E-07
chr12	25245350	G	T	KRAS	0.13%	0.17	2.40177E-06
chr12	25245350	G	A	KRAS	0.13%	0.11	1.42788E-07
chr12	25245351	G	C	KRAS	0.13%	0.11	1.18801E-07
chr12	25245351	G	A	KRAS	0.13%	0.08	4.36218E-08

chr12	25245351	G	T	KRAS	0.13%	0.12	6.32549E-07
chr12	25227341	A	C	KRAS	0.13%	0.13	1.57217E-07
chr12	25227342	A	T	KRAS	0.13%	0.11	6.56882E-08
chr15	66436825	C	T	MEK1	0.13%	0.10	1.6522E-07
chr7	116783374	T	G	MET	0.10%	0.11	1.18091E-07
chr9	136504892	A	G	NOTCH	0.13%	0.12	1.35229E-06
chr1	114713907	A	T	NRAS	0.13%	0.11	5.12847E-08
chr1	114713909	C	A	NRAS	0.13%	0.12	8.45867E-09
chr1	114713908	A	T	NRAS	0.13%	0.09	3.86302E-08
chr1	114713908	A	G	NRAS	0.13%	0.18	2.29329E-07
chr4	54285926	A	T	PDGFRA	0.13%	0.22	6.73089E-07
chr3	179218294	G	A	PIK3CA	0.13%	0.07	2.65636E-08
chr3	179218303	G	A	PIK3CA	0.13%	0.05	6.84847E-08
					Min	0.03	
					Max	0.26	
					Average	0.12	
					Variance of all Allele Frequency Average	0.002062299	

Supplementary Table 5. Detected and expected allele frequencies for 0.13% reference mutations with 20.0 ng of reference material. Individual mutation averages and variances are of 3 biological replicates.

Pilot Study	Sample ID	Cancer Type	Sample Type	Sequencing Instrument	PE Reads	PE Reads after trimming	Genome Mapping Rate (%)	On-Target Reads	Total Discarded Reads (%)	Average Molecular Depth Across All Primers	Average UMI Family Duplication Rate
1	1	Colon	FFPE	MiSeq	985,819	785,015	94.3	666,022	32.44	120	19
1	2	Colon	FFPE	MiSeq	1,848,004	1,784,784	99.5	1,620,782	12.30	350	16
1	3	Colon	FFPE	MiSeq	2,604,889	2,559,946	99.4	2,315,415	11.11	800	10
1	4	Colon	FFPE	MiSeq	1,074,834	869,123	94.1	747,384	30.47	100	26
1	5	Colon	FFPE	MiSeq	840,949	714,666	96.3	611,913	27.24	105	20
1	6	Colon	FFPE	MiSeq	937,313	849,723	96.8	756,101	19.33	130	20
1	7	Colon	FFPE	MiSeq	897,696	803,139	96.2	703,087	21.68	150	16
1	8	Colon	FFPE	MiSeq	1,059,402	966,072	97.3	858,246	18.99	125	23
1	9	Colon	FFPE	MiSeq	1,014,539	925,284	97.6	824,192	18.76	145	19
1	10	Colon	FFPE	MiSeq	1,008,351	957,313	98.4	878,244	12.9	165	18
1	11	Colon	FFPE	MiSeq	1,105,446	1,004,834	97.0	914172	17.3	130	24
1	12	Colon	FFPE	MiSeq	925,498	688,384	89.3	554830	40.05	120	16
1	13	Colon	FFPE	MiSeq	5,202,032	5,074,762	99.2	4716933	9.33	560	29
1	14	Colon	FFPE	MiSeq	1,048,658	948,665	98.3	859501	18.04	360	8
2	1	Lung	cfDNA	NextSeq	5,725,286	5,588,152	99.925	5,113,228	10.69	623	23
2	2	Lung	cfDNA	NextSeq	5,962,896	5,823,697	99.93	5,256,244	11.852	712	20
2	3	Lung	cfDNA	NextSeq	5,916,846	5,667,624	99.92	5,157,748	12.82	920	16
2	4 rep 1	Lung	cfDNA	NextSeq	6,439,427	6,299,592	99.95	5,598,895	13.05	1432	11
2	4 rep 2	Lung	cfDNA	NextSeq	5,422,449	5,300,468	99.935	4,729,110	12.79	1378	10
2	4 rep 3	Lung	cfDNA	NextSeq	5,694,393	5,572,667	99.95	4,960,578	12.89	1394	10
2	5	Lung	cfDNA	NextSeq	6,211,345	6,076,756	99.94	5,510,784	12.89	885	17
2	6	Lung	cfDNA	NextSeq	5,946,534	5,815,572	99.925	5,250,403	11.28	1272	11
2	7	Lung	cfDNA	NextSeq	5,591,169	5,480,830	99.95	4,998,118	11.71	1393	10

2	8	Lung	FFPE	NextSeq	5,911,637	5,640,270	99.92	5,156,788	10.61	2605	5
2	9	Lung	FFPE	NextSeq	5,524,161	5,249,362	99.9	4,648,396	12.78	2027	6
2	10	Lung	FFPE	NextSeq	5,248,340	4,814,414	99.87	4,118,638	15.85	1386	8
2	11 rep 1	Lung	FFPE	NextSeq	6,183,401	5,908,447	99.64	5,380,817	21.52	2320	6
2	11 rep 2	Lung	FFPE	NextSeq	6,892,037	6,580,624	99.92	5,942,841	13.77	2347	7
2	11 rep 3	Lung	FFPE	NextSeq	5,516,677	5,254,466	99.91	4,732,011	14.22	2200	6
2	12	Lung	FFPE	NextSeq	5,305,599	4,954,218	99.91	4,365,513	17.71	2355	5
2	13	Lung	FFPE	NextSeq	4,889,296	4,644,891	99.915	4,101,554	16.11	2584	4
3	1	Lung	FFPE	HiSeq	14,602,226	14,318,563	99.9	10,957,299	24.96	699	14
3		Lung	cfDNA	HiSeq	39,846,459	39,506,756	99.8	35,710,156	10.38	341	91
3	2	Lung	FFPE	MiSeq	7,416,038	7,364,548	99.47	6,245,973	15.78	502	11
3		Lung	cfDNA	MiSeq	3,287,358	3,270,428	99.49	2,877,043	12.48	318	8
3	3	Lung	FFPE	MiSeq	6,725,536	6,680,534	99.24	5,821,878	13.44	317	16
3		Lung	cfDNA	MiSeq	3,368,818	3,339,231	99.07	2,799,226	16.91	157	16
3	4	Lung	FFPE	HiSeq + MiSeq	58,845,179	58,253,137	99.92	49,768,512	15.42	1302	33
3		Lung	cfDNA	HiSeq + MiSeq	65,825,543	64,980,927	99.57	55,873,230	15.12	404	121
3	5	Lung	FFPE	HiSeq	13,025,985	12,840,684	99.82	10,510,952	19.31	736	12
3		Lung	cfDNA	HiSeq	40,727,884	40,060,560	99.91	33,649,673	17.38	402	73
3	6	Lung	FFPE	MiSeq	3,388,633	3,362,384	99.19	2,830,684	16.67	341	7
3		Lung	cfDNA	MiSeq	6,993,570	6,948,773	99.22	6,075,001	13.13	192	28
3	7	Lung	FFPE	MiSeq	6,395,028	6,355,166	99.25	5,524,430	13.61	710	7
3		Lung	cfDNA	MiSeq	3,288,506	3,259,063	99.22	2,723,688	17.18	322	7
3	8	Lung	FFPE	HiSeq	13,399,391	13,138,464	99.94	10,641,074	20.59	494	19
3		Lung	cfDNA	HiSeq	42,230,425	41,782,236	99.92	37,666,685	10.81	399	82
3	9	Lung	FFPE	HiSeq + MiSeq	68,031,679	67,337,022	99.72	58,141,656	14.53	1365	37

3	9	Lung	cfDNA	HiSeq + MiSeq	83,936,128	83,097,157	99.57	72,121,859	14.08	458	137
3	10	Lung	FFPE	HiSeq	14,949,016	14,691,872	99.89	11,199,540	25.08	686	14
3		Lung	cfDNA	HiSeq	40,671,065	40,325,796	99.91	36,519,040	10.21	419	76
3	11	Lung	FFPE	HiSeq	13,136,302	12,902,357	99.83	9,768,618	25.64	613	14
3		Lung	cfDNA	HiSeq	61,735,997	60,727,836	99.93	52,592,264	14.81	623	74
3	12	Lung	FFPE	HiSeq	16,275,543	15,985,473	99.94	13,046,422	19.84	569	20
3		Lung	cfDNA	HiSeq	30,399,924	29,972,469	99.6	24,574,427	19.16	280	77
3	13	Lung	FFPE	HiSeq	13,354,101	13,055,028	99.3	10,562,155	20.90	507	18
3		Lung	cfDNA	HiSeq	36,594,461	36,099,848	99.79	29,872,624	18.37	280	93
3	15	Lung	FFPE	HiSeq	12,833,416	12,627,086	99.85	9,911,381	22.77	640	14
3		Lung	cfDNA	HiSeq	48,343,010	47,583,782	99.92	40,649,363	15.91	512	69
3	14	Lung	FFPE	MiSeq	8,502,361	8,440,206	99.1	7,099,356	16.50	595	10
3		Lung	cfDNA	MiSeq	11,378,163	11,312,717	99.1	9,755,408	14.26	330	26
3	16	Lung	FFPE	MiSeq	8,038,419	8,038,419	99.32	6,708,126	16.55	691	8
3		Lung	cfDNA	MiSeq	2,973,720	2,973,720	99.46	2,532,400	14.84	364	6
3	17	Lung	FFPE	HiSeq	11,109,068	11,109,068	99.93	9,025,478	18.76	591	13
3		Lung	cfDNA	HiSeq	59,335,783	59,335,783	99.96	50,337,625	15.16	977	45
3	18	Lung	FFPE	HiSeq + MiSeq	83,910,995	82,923,357	99.49	67,008,596	20.14	1461	40
3		Lung	cfDNA	HiSeq + MiSeq	79,650,362	78,759,361	99.53	68,040,314	14.58	808	73
3	19	Lung	FFPE	MiSeq	3,672,913	3,644,275	99.02	3,041,597	17.19	377	7
3		Lung	cfDNA	MiSeq	7,792,509	7,738,808	98.94	6,720,939	13.75	169	35
3	20	Lung	FFPE	HiSeq	12,456,063	12,193,429	99.94	9,855,467	20.88	504	17
3		Lung	cfDNA	HiSeq	38,831,522	38,292,615	99.78	31,656,504	18.48	284	97

Supplementary Table 6. Details of all sequencing for pilot studies 1, 2 and 3.

Sample ID	Cancer Type	Sample Type	Gene	Chromosome	Start position	End position	Reference allele sequence	Alternative allele sequence	Depth	Alt Depth	AF (%)	Alternative Technology AF (%)
1	Colon	FFPE	KRAS	chr12	25245350	25245350	C	T	82	7	8.5	WT
1	Colon	FFPE	NRAS	chr1	114713909	114713909	G	T	172	92	53.5	56.3
1	Colon	FFPE	PIK3CA	chr3	179218303	179218303	G	A	56	37	66.1	70
2	Colon	FFPE	PIK3CA	chr3	179218294	179218294	G	A	400	119	29.8	24.2
3	Colon	FFPE	KRAS	chr12	25227341	25227341	T	G	1123	474	42.2	47.6
4	Colon	FFPE	KRAS	chr12	25227342	25227342	T	A	67	16	23.9	42.5
4	Colon	FFPE	PIK3CA	chr3	179218294	179218294	G	A	26	2	7.7	WT
5	Colon	FFPE	KRAS	chr12	25245350	25245351	CC	AG	89	35	39.3	30.3
7	Colon	FFPE	KRAS	chr12	25225628	25225628	C	T	335	95	28.4	34
7	Colon	FFPE	PIK3CA	chr3	179234297	179234297	A	G	164	23	14.0	13
8	Colon	FFPE	KRAS	chr12	25245347	25245347	C	T	128	73	57.0	93
9	Colon	FFPE	BRAF	chr7	140753336	140753336	A	T	215	30	14.0	19.8
9	Colon	FFPE	TP53	chr17	7,670,716	7,670,716	G	A	66	14	21.0	16
10	Colon	FFPE	BRAF	chr7	140753336	140753336	A	T	239	74	31.0	34.7
11	Colon	FFPE	KRAS	chr12	25225628	25225628	C	T	246	102	41.5	34
11	Colon	FFPE	PIK3CA	chr3	179234297	179234297	A	G	88	41	46.6	48
11	Colon	FFPE	TP53	chr17	7675119	7675119	C	T	95	29	31.0	30
12	Colon	FFPE	KRAS	chr12	25245350	25245350	C	T	38	5	13.2	8.9
12	Colon	FFPE	PIK3CA	chr3	179218294	179218294	G	A	27	2	7.4	WT
13	Colon	FFPE	KRAS	chr12	25245350	25245350	C	A	1213	294	24.2	22
14	Colon	FFPE	PIK3CA	chr3	179218303	179218303	G	A	306	158	51.6	47
14	Colon	FFPE	TP53	chr17	7675208	7675208	G	A	388	18	18.0	17

Supplementary Table 7. A list of variants from clinical samples for pilot study 1.

Sample ID	Cancer Type	Sample Type	Gene	Chr	Start position	End position	Reference allele sequence	Alternative allele sequence	Depth	Alt Depth	AF (%)	Alternative Technology AF (%)
1	Lung	cfDNA	EGFR	chr7	55191822	55191822	T	G	675	33	4.9	2
2	Lung	cfDNA	EGFR	chr7	55181378	55181378	C	T	920	10	1.1	47.8
2	Lung	cfDNA	EGFR	chr7	55191822	55191822	T	G	1091	11	1.0	44.5
3	Lung	cfDNA	EGFR	chr7	55174771	55174786	AGGAATTAAGAGAAGC	A	1024	334	32.6	35.7
3	Lung	cfDNA	EGFR	chr7	55181378	55181378	C	T	1243	240	19.3	18.9
4 rep 1	Lung	cfDNA	EGFR	chr7	55191822	55191822	T	G	1608	706	43.9	33.2
4 rep 1	Lung	cfDNA	KRAS	chr12	25245351	25245351	C	T	2645	440	16.6	15.3
4 rep 2	Lung	cfDNA	EGFR	chr7	55191822	55191822	T	G	1549	627	40.5	33.2
4 rep 2	Lung	cfDNA	KRAS	chr12	25245351	25245351	C	T	2582	456	17.7	15.3
4 rep 3	Lung	cfDNA	EGFR	chr7	55191822	55191822	T	G	1558	642	41.2	33.2
4 rep 3	Lung	cfDNA	KRAS	chr12	25245351	25245351	C	T	2615	445	17.2	15.3
5	Lung	cfDNA	EGFR	chr7	55174776	55174794	TTAAGAGAAGCAACATCTC	T	862	32	3.7	2.4
6	Lung	cfDNA	EGFR	chr7	55174772	55174792	GGAATTAAGAGAAGCAACATC	AAT	1183	36	3.0	4.7
7	Lung	cfDNA	EGFR	chr7	55174771	55174786	AGGAATTAAGAGAAGC	A	1968	779	39.6	19.7
9	Lung	FFPE	EGFR	chr7	55174776	55174788	TTAAGAGAAGCAA	C	2453	830	33.8	70
10	Lung	FFPE	EGFR	chr7	55174771	55174786	AGGAATTAAGAGAAGC	A	1440	393	27.3	33.8
11	Lung	FFPE	EGFR	chr7	55191822	55191822	T	G	958	384	40.1	21.2
12 rep 1	Lung	FFPE	NRAS	chr1	114713909	114713909	G	T	2847	725	25.0	20.9
12 rep 2	Lung	FFPE	NRAS	chr1	114713909	114713909	G	T	2760	729	26.4	20.9
12 rep 3	Lung	FFPE	NRAS	chr1	114713909	114713909	G	T	2625	669	24.5	20.9
13	Lung	FFPE	APC	chr5	112838329	112838329	T	A	97	17	17.5	9.9
13	Lung	FFPE	FBXW7	chr4	152324294	152324294	G	A	262	16	8.1	7
13	Lung	FFPE	FBXW7	chr4	152326214	152326214	C	T	2957	242	6.1	7.8
13	Lung	FFPE	KRAS	chr12	25245351	25245351	C	A	3202	265	8.3	7.8
14	Lung	FFPE	EGFR	chr7	55191822	55191822	T	G	5727	1816	31.0	32

Supplementary Table 8. A list of variants from clinical samples for pilot study 2.

								ATOM-Seq			Alternative Technology			
Sample ID	Sample Type	Gene	Chr	Start position	End position	Reference allele sequence	Alternative allele sequence	Depth	Alt Depth	AF (%)	Depth	Alt Depth	AF (%)	Using UMI
1	FFPE	FGFR1	chr8	38414245	38414245	C	G	57	4	7.0	Region Not Covered by Panel			
1	cfDNA							57	0	0.0				
2	FFPE	ERBB2	chr17	39724748	39724748	T	TGGGCTCCCC	1759	325	18.5	388	97	25.0	yes
2	cfDNA							2449	0	0.0	Sample Not Tested			
3	FFPE	TP53	chr17	7673803	7673803	G	A	149	116	77.9	3087	1613	52.25	no
3	cfDNA							84	3	3.6	Sample Not Tested			
4	FFPE	TP53	chr17	7674917	7674917	T	C	767	58	7.6	164	13	7.93	yes
4	cfDNA							159	0	0.0	237	0	0	yes
4	FFPE	MAP2K1	chr15	66436755	66436761	CTGGAGA	C	238	29	12.2	458	30	6.5	yes
4	cfDNA							54	2	3.7	232	0	0	yes
5	FFPE	TP53	chr17	7675122	7675122	T	A	785	246	31.3	3819	1316	34.46	no
5	cfDNA							877	5	0.6	Sample Not Tested			
5	FFPE	KEAP1	chr19	10491620	10491620	C	A	1015	389	38.3	Region Not Covered by Panel			
5	cfDNA							715	3	0.4				
6	FFPE	RB1	chr13	48459770	48459770	G	A	117	72	61.5	Region Not Covered by Panel			
6	cfDNA							120	8	6.7				
6	FFPE	TP53	chr17	7674885	7674885	C	T	301	205	68.1	5591	4115	73.6	no
6	cfDNA							645	72	11.2	Sample Not Tested			
7	FFPE	TP53	chr17	7674872	7674872	T	C	428	116	27.1	4098	1265	30.87	no
7	cfDNA							553	5	0.9	Sample Not Tested			
7	FFPE	TP53	chr17	7675122	7675122	T	C	1052	438	41.6	233	69	29.61	no
7	cfDNA							1149	33	2.9	Sample Not Tested			
8	FFPE	ERBB3	chr12	56088574	56088574	C	T	300	54	18.0	Region Not Covered by Panel			
8	cfDNA							202	0	0.0				
8	FFPE	TP53	chr17	7675135	7675139	GGCGC	G	816	269	33.0	3563	1362	38.2	no

8	cfDNA							715	0	0.0	Sample Not Tested			
9	FFPE	MSH2	chr2	47410292	47410292	G	T	329	21	6.4	460	92	20	yes
9	cfDNA							280	3	1.1	67	3	4.48	yes
9	FFPE	KEAP1	chr19	10491944	10491944	G	A	126	19	15.1	280	69	24.45	yes
9	cfDNA							114	1	0.9	44	0	0	yes
9	FFPE	TP53	chr17	7673802	7673802	C	T	591	178	30.1	280	68	24.29	yes
9	cfDNA							277	6	2.2	68	0	0	yes
10	FFPE	TP53	chr17	7675088	7675088	C	T	789	68	8.6	7234	653	9.0	no
10	cfDNA							272	0	0.0	Sample Not Tested			
10	FFPE	TP53	chr17	7674241	7674241	G	C	808	89	11.0	13085	1346	10.3	no
10	cfDNA							3044	6	0.2	Sample Not Tested			
10	FFPE	PIK3CA	chr3	179218307	179218307	A	C	1707	519	30.4	7274	2142	29.44	no
10	cfDNA							440	7	1.6	Sample Not Tested			
11	FFPE	TP53	chr17	7674247	7674247	T	C	607	66	10.9	Region Not Covered by Panel			
11	cfDNA							2767	0	0.0				
11	FFPE	JAK3	chr19	17834899	17834899	C	G	125	17	13.6	Region Not Covered by Panel			
11	cfDNA							155	14	9.0				
11	FFPE	BRCA1	chr17	43093010	43093010	G	A	114	25	21.9	Region Not Covered by Panel			
11	cfDNA							287	109	38.0				
12	FFPE	TSC2	chr16	2064406	2064406	C	T	342	35	10.2	Region Not Covered by Panel			
12	cfDNA							1293	0	0.0				
12	FFPE	RB1	chr13	48465204	48465205	GC	G	263	64	24.3	Region Not Covered by Panel			
12	cfDNA							27	2	7.4				
12	FFPE	TP53	chr17	7676381	7676381	C	A	184	74	40.2	8559	3320	38.78	no
12	cfDNA							190	1	0.5	Sample Not Tested			
13	FFPE	TP53	chr17	7675994	7675994	C	A	711	297	41.8	1817	851	46.86	no
13	cfDNA							264	5	1.9	Sample Not Tested			
13	FFPE	BRAF	chr7	140749350	140749350	T	C	536	229	42.7	Region Not Covered by Panel			
13	cfDNA							110	0	0.0				

13	FFPE	KEAP1	chr19	10499551	10499551	C	A	150	65	43.3	Region Not Covered by Panel			
13	cfDNA							0	0	0.0				
14	FFPE	CTNNB1	chr3	41235749	41235749	T	G	871	75	8.6	Region Not Covered by Panel			
14	cfDNA							492	3	0.6				
14	FFPE	TP53	chr17	7670694	7670694	C	A	652	164	25.2	824	164	19.9	no
14	cfDNA							580	8	1.4	Sample Not Tested			
14	FFPE	DMD1	chrX	32501785	32501785	C	A	330	85	25.8	Region Not Covered by Panel			
14	cfDNA							104	0	0.0				
15	FFPE	IDH1	chr2	208248468	208248468	G	A	890	143	16.1	Region Not Covered by Panel			
15	cfDNA							241	58	24.1				
15	FFPE	KEAP1	chr19	10491954	10491954	C	T	57	25	43.9	Region Not Covered by Panel			
15	cfDNA							247	4	1.6				
15	FFPE	KEAP1	chr19	10489702	10489702	C	G	90	70	77.8	Region Not Covered by Panel			
15	cfDNA							190	13	6.8				
15	FFPE	TP53	chr17	7675139	7675139	C	A	1005	838	83.4	2901	2517	86.76	no
15	cfDNA							1705	120	7.0	Sample Not Tested			
16	FFPE	ATM	chr11	108299779	108299779	A	C	871	144	16.5	Region Not Covered by Panel			
16	cfDNA							751	369	49.1				
16	FFPE	MSH6	chr2	47803442	47803442	C	T	3018	1055	35.0	7231	2326	32.2	no
16	cfDNA							2524	8	0.3	Sample Not Tested			
16	FFPE	BRCA2	chr13	32337717	32337745	CAGGAAGTCAGTTTG AATTTACTCAGTTT	C	2045	817	40.0	Region Not Covered by Panel			
16	cfDNA							406	2	0.5				
16	FFPE	TP53	chr17	7675076	7675076	T	A	743	326	43.9	7641	4178	54.68	no
16	cfDNA							775	1	0.1	Sample Not Tested			
17	FFPE	MAP2K2	chr19	4117520	4117520	T	C	462	55	11.90	Region Not Covered by Panel			
17	cfDNA							1422	0	0.0				
17	FFPE	CSF1R	chr5	150054182	150054203	CGCTGCCACCG CTTCTGCTGCT	C	22	4	18.2	Region Not Covered by Panel			
17	cfDNA							50	11	22.0				
17	FFPE	KEAP1	chr19	10491944	10491944	G	A	119	26	21.8	Region Not Covered by Panel			

17	cfDNA							806	25	3.1				
17	FFPE	TP53	chr17	7674216	7674216	C	G	1304	315	24.2	6192	2023	32.67	no
17	cfDNA							3269	74	2.3	Sample Not Tested			
17	FFPE	STK11	chr19	1220494	1220494	G	T	400	121	30.3	Region Not Covered by Panel			
17	cfDNA							633	17	2.7				
18	FFPE	MAP2K2	chr19	4101064	4101064	G	T	120	16	13.3	Region Not Covered by Panel			
18	cfDNA							357	17	4.8				
18	FFPE	ROS1	chr6	117317224	117317224	C	A	341	58	17.0	1910	339	17.7	yes
18	cfDNA							195	0	0.0	183	0	0	yes
18	FFPE	CDKN2A	chr9	21971187	21971187	G	A	900	231	25.7	1020	433	42.5	yes
18	cfDNA							505	2	0.4	234	0	0	yes
18	FFPE	SMARC4	chr19	11033317	11033317	C	T	667	180	27.0	698	335	48.0	yes
18	cfDNA							1261	15	1.2	118	2	1.69	yes
18	FFPE	TP53	chr17	7675085	7675085	C	A	2405	694	28.9	308	117	38.0	yes
18	cfDNA							2112	11	0.5	176	0	0	yes

Supplementary Table 9. A list of variants from clinical samples for pilot study 3 using paired lung cancer samples. Samples processed by an alternative technology using UMI were processed using a QIAseq targeted DNA Panel all others were processed using an Illumina trusight panel.

Input Mass (ng)	Material Type	Sequencing Instrument	PE Reads	PE Reads after trimming	On-Target Reads	Total Discarded Reads (%)
100	FFPE-RNA Reference	MiSeq	784,799	782,706	722,615	7.92
13.65	Cell-free Total Nucleic Acids (DNA+RNA)	MiSeq	902,066	899,764	837,723	7.13

Supplementary Table 10. Details of sequencing for RNA reference standards and cell-free Total Nucleic Acids.

Pan Caner Panel Targets	Lung Panel Targets	Colorectal Panel Targets	Custom Fusion Panel Targets
ABL1	AMER1	AMER1	ABL
AKT1	APC	APC	ALK
ALK	ARAF	ARAF	BRAF
AMER1	BRAF	BRAF	EGFR
APC	CDKN2A	CTNNB1	ERBB2
AR	DMD	DMD	ERG
ARAF	EGFR	EP300	ETV6
ARID1A	EP300	ERBB3	EWSR1
ATM	ERBB3	FBWX7	FGFR1
BRAF	FBXW7	FGFR4	FGFR2
BRCA1	GNAS	GNAS	FGFR3
BRCA2	HRAS	HRAS	FGR
CASP8	KEAP1	KRAS	JAK1
CCND1	KRAS	MAP2K1	JAK2
CCND2	MAP2K1	MAP2K2	JAK3
CCND3	MAP2K2	NRAS	NTRK1
CDH1	NRAS	PDGFRA	NTRK3
CDK4	PDGFA	PIK3CA	PDGFRB
CDK6	PIK3CA	ROS1	RAF
CDKN2A	SMAD4	SMAD4	RARA
CHEK2	STK11	TCF7L2	RET
CSF1R	TCF7L2	TP53	ROS1
CTNNB1	TP53		
DDR2			
DMD			
EGFR			
EP300			
ERBB2			
ERBB3			
ERBB4			
ESR1			
EZH2			
FBXW7			
FGFR1			
FGFR2			
FGFR3			
FGFR4			
FLT3			
GATA3			
GNA11			
GNAQ			
GNAS			
HNF1A			

HRAS			
IDH1			
IDH2			
JAK2			
JAK3			
KDM6A			
KDR			
KEAP1			
KIT			
KLF5			
KRAS			
MAP2K1			
MAP2K2			
MET			
MGA			
MLH1			
MPL			
MSH2			
MSH6			
MTOR			
MYC			
NF1			
NFE2L2			
NOTCH1			
NPM1			
NRAS			
NTRK1			
NTRK3			
PDGFRA			
PIK3CA			
PTCH1			
PTEN			
PTPN11			
RAF1			
RB1			
RBM10			
RET			
RHOA			
RIT1			
RNF43			
ROS1			
SETD2			
SF3B1			
SMAD2			

SMAD4			
SMARCA4			
SMARCB1			
SMO			
SRC			
STK11			
TCF7L2			
TP53			
TSC1			
TSC2			
UA2F1			
VHL			
ZFP36L2			

Supplementary Table 11. A list of target genes present in all primer panels.
